# 2-Methyl-3-(2-methyl­phen­yl)-4-oxo-3,4-dihydro­quinazolin-8-yl 4-methyl­benzoate

**DOI:** 10.1107/S1600536812006265

**Published:** 2012-02-17

**Authors:** Adel S. El-Azab, Alaa A.-M. Abdel-Aziz, Seik Weng Ng, Edward R. T. Tiekink

**Affiliations:** a Department of Pharmaceutical Chemistry, College of Pharmacy, King Saud University, Riyadh 11451, Saudi Arabia; b Department of Organic Chemistry, Faculty of Pharmacy, Al-Azhar University, Cairo 11884, Egypt; c Department of Medicinal Chemistry, Faculty of Pharmacy, University of Mansoura, Mansoura 35516, Egypt; d Department of Chemistry, University of Malaya, 50603 Kuala Lumpur, Malaysia; e Chemistry Department, Faculty of Science, King Abdulaziz University, PO Box 80203 Jeddah, Saudi Arabia

## Abstract

In the title quinazolin-4-one derivative, C_24_H_20_N_2_O_3_, both the 4-methyl­benzoate [dihedral angle = 83.90 (9)°] and 2-tolyl [87.88 (9)°] groups are almost orthogonal to the central fused ring system. These aryl groups are oriented towards the quinazolin-4-one-bound methyl group. In the crystal, mol­ecules are connected into a three-dimensional architecture by C—H⋯O, C—H⋯π and π–π [ring centroid-to-centroid separation = 3.6458 (13) Å] inter­actions.

## Related literature

For the pharmacological activity of substituted quinazoline-4(3*H*)-ones, see: El-Azab & ElTahir (2012 ▶); El-Azab *et al.* (2011 ▶); Al-Omary *et al.* (2010 ▶); Al-Obaid *et al.* (2009 ▶); Aziza *et al.* (1996 ▶). For the synthesis and evaluation of the anti­convulsant activity of the title compound, see: El-Azab *et al.* (2010 ▶). For the structure of the benzoate derivative, see: El-Azab *et al.* (2012 ▶).
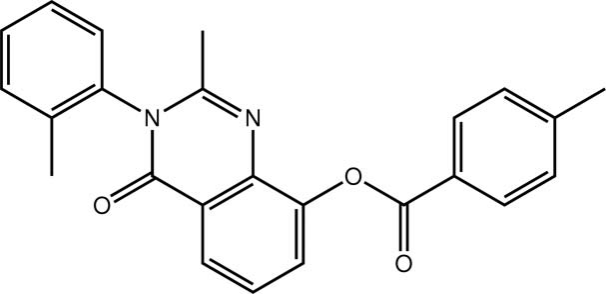


## Experimental

### 

#### Crystal data

C_24_H_20_N_2_O_3_
*M* *_r_* = 384.42
Monoclinic, 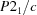

*a* = 18.8216 (5) Å
*b* = 7.6332 (2) Å
*c* = 13.3092 (3) Å
β = 97.286 (2)°
*V* = 1896.68 (8) Å^3^
*Z* = 4
Cu *K*α radiation
μ = 0.72 mm^−1^
*T* = 100 K
0.25 × 0.20 × 0.15 mm

#### Data collection

Agilent SuperNova Dual diffractometer with an Atlas detector
Absorption correction: multi-scan (*CrysAlis PRO*; Agilent, 2011 ▶) *T*_min_ = 0.755, *T*_max_ = 1.000
7966 measured reflections
3883 independent reflections
3478 reflections with *I* > 2σ(*I*)
*R*_int_ = 0.027

#### Refinement

*R*[*F* ^2^ > 2σ(*F* ^2^)] = 0.067
*wR*(*F* ^2^) = 0.186
*S* = 1.06
3883 reflections
265 parameters
H-atom parameters constrained
Δρ_max_ = 1.09 e Å^−3^
Δρ_min_ = −0.33 e Å^−3^

### 

Data collection: *CrysAlis PRO* (Agilent, 2011 ▶); cell refinement: *CrysAlis PRO*; data reduction: *CrysAlis PRO*; program(s) used to solve structure: *SHELXS97* (Sheldrick, 2008 ▶); program(s) used to refine structure: *SHELXL97* (Sheldrick, 2008 ▶); molecular graphics: *ORTEP-3* (Farrugia, 1997 ▶) and *DIAMOND* (Brandenburg, 2006 ▶); software used to prepare material for publication: *publCIF* (Westrip, 2010 ▶).

## Supplementary Material

Crystal structure: contains datablock(s) global, I. DOI: 10.1107/S1600536812006265/hb6636sup1.cif

Structure factors: contains datablock(s) I. DOI: 10.1107/S1600536812006265/hb6636Isup2.hkl

Supplementary material file. DOI: 10.1107/S1600536812006265/hb6636Isup3.cml

Additional supplementary materials:  crystallographic information; 3D view; checkCIF report

## Figures and Tables

**Table 1:** Hydrogen-bond geometry (Å, °) *Cg*1 is the centroid of the C18–C23 benzene ring.

*D*—H⋯*A*	*D*—H	H⋯*A*	*D*⋯*A*	*D*—H⋯*A*
C17—H17*C*⋯O2^i^	0.98	2.55	3.434 (3)	150
C21—H21⋯O3^ii^	0.95	2.47	3.299 (3)	146
C12—H12⋯*Cg*1^iii^	0.95	2.79	3.658 (2)	153

Symmetry codes: (i) 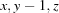; (ii) 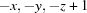; (iii) 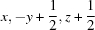.

**Fig. 1.**
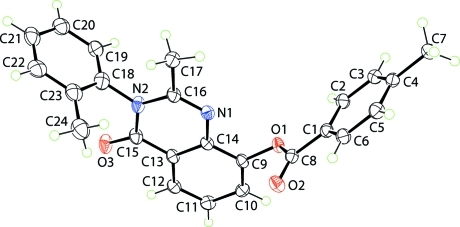
The molecular structure of (I) showing displacement ellipsoids at the 50% probability level.

**Fig. 2.**
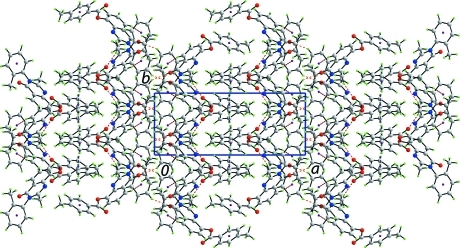
A view in projection down the c axis of the unit-cell contents for (I). The C—H···O, C—H···π and π–π interactions are shown as orange, brown and purple dashed lines, respectively.

## supplementary crystallographic information

### Comment

The title compound, (I), a methaqualone analogue, was recently synthesized and evaluated for its anti-convulsant activity (El-Azab *et al.*, 2010). Herein, the crystal structure determination of 3,4-dihydro-2-methyl-3-(2-methylphenyl)-4-oxoquinazolin-8-yl 4-methylbenzoate (I) is reported. These studies were motivated by the observation that substituted quinazoline-4(3*H*)-ones are known to display various biological activities (El-Azab & ElTahir, 2012; El-Azab *et al.*, 2011; El-Azab *et al.*, 2010; Al-Omary *et al.*, 2010; Al-Obaid *et al.*, 2009; Aziza *et al.*, 1996).

In (I), Fig. 1, the carboxylate residue is co-planar to the benzene ring to which it is connected as seen in the value of the C2—C1—C8—O1 torsion angle -3.8 (3)°. With respect to the central quinazolin-4-one fused ring system [r.m.s. deviation = 0.045 Å for the 10 atoms], both the 4-methylbenzoate and 2-tolyl groups are orthogonal: the dihedral angles between the central plane and six-membered rings being 83.90 (9) and 87.88 (9)°, respectively. Both aryl substituents are orientated towards the methyl group bound to the quinazolin-4-one system and the dihedral angle between the two six-membered rings is 77.04 (11)°. The molecular structure resembles closely that of the benzoate derivative (El-Azab *et al.*, 2012).

In the crystal packing, C—H···O [involving both carbonyl-O atoms] and C—H···π [involving the (C18···C23) benzene ring] interactions, Table 1, lead to the formation of layers in the *bc* plane. These interdigitate to allow for the formation of π–π interactions between the 4-methylbenzoate rings [ring centroid···centroid separation = 3.6458 (13) Å between centrosymmetrically related (C1–C6) rings; symmetry operation: 1 - *x*, 2 - *y*, 1 - *z*]. The combination of intermolecular interactions leads to a three-dimensional architecture, Fig. 2.

### Experimental

A mixture of 8-hydroxymethaqualone (532 mg, 0.002 *M*) and 4-methylbenzoyl chloride (325 mg, 0.0021 mmol) in 15 ml pyridine was stirred at room temperature for 12 h. The solvent was removed under reduced pressure, and the residue was triturated with water and filtered. The solid obtained was dried and recrystallized from EtOH to yield colourless prisms. m.p. 465–467 K. Yield: 95%. ^1^H NMR (500 MHz, CDCl_3_): δ = 8.25–8.22 (m, 3H), 7.64 (d, 1H, J = 7.5 Hz), 7.52 (t, 1H, J = 7.5 Hz), 7.43–7.28 (m, 5H), 7.15 (d, 1H, J = 7.5 Hz), 2.49 (s, 3H), 2.15 (s, 3H), 2.11 (s, 3H) p.p.m.. ^13^C NMR (CDCl_3_): δ = 17.4, 21.8, 24.3, 120.7, 124.9, 126.4, 126.7, 127.4, 127.6, 127.9, 129.3, 129.6, 130.5, 131.5, 135.4, 136.8, 141.0, 144.5, 146.3, 154.7, 161.2, 165.3 p.p.m.. MS (70 eV): m/*z* = 384.

### Refinement

Carbon-bound H atoms were placed in calculated positions [C—H = 0.95 to 0.98 Å, *U*_iso_(H) = 1.2–1.5*U*_eq_(C)] and were included in the refinement in the riding model approximation. The maximum and minimum residual electron density peaks of 1.09 and 0.33 e Å^-3^, respectively, were located 0.92 Å and 0.56 Å from the C18 and C23 atoms, respectively.

### Crystal data

**Table:**

C_24_H_20_N_2_O_3_	*F*(000) = 808
*M**_r_* = 384.42	*D*_x_ = 1.346 Mg m^−^^3^
Monoclinic, *P*2_1_/*c*	Cu *K*α radiation, λ = 1.5418 Å
Hall symbol: -P 2ybc	Cell parameters from 3857 reflections
*a* = 18.8216 (5) Å	θ = 3.4–76.5°
*b* = 7.6332 (2) Å	µ = 0.72 mm^−^^1^
*c* = 13.3092 (3) Å	*T* = 100 K
β = 97.286 (2)°	Prism, colourless
*V* = 1896.68 (8) Å^3^	0.25 × 0.20 × 0.15 mm
*Z* = 4	

### Data collection

**Table:**

Agilent SuperNova Dual diffractometer with an Atlas detector	3883 independent reflections
Radiation source: SuperNova (Cu) X-ray Source	3478 reflections with *I* > 2σ(*I*)
Mirror monochromator	*R*_int_ = 0.027
Detector resolution: 10.4041 pixels mm^-1^	θ_max_ = 76.7°, θ_min_ = 4.7°
ω scans	*h* = −23→23
Absorption correction: multi-scan (*CrysAlis PRO*; Agilent, 2011)	*k* = −9→9
*T*_min_ = 0.755, *T*_max_ = 1.000	*l* = −16→8
7966 measured reflections	

### Refinement

**Table:**

Refinement on *F*^2^	Primary atom site location: structure-invariant direct methods
Least-squares matrix: full	Secondary atom site location: difference Fourier map
*R*[*F*^2^ > 2σ(*F*^2^)] = 0.067	Hydrogen site location: inferred from neighbouring sites
*wR*(*F*^2^) = 0.186	H-atom parameters constrained
*S* = 1.06	*w* = 1/[σ^2^(*F*_o_^2^) + (0.0962*P*)^2^ + 2.188*P*] where *P* = (*F*_o_^2^ + 2*F*_c_^2^)/3
3883 reflections	(Δ/σ)_max_ < 0.001
265 parameters	Δρ_max_ = 1.09 e Å^−^^3^
0 restraints	Δρ_min_ = −0.33 e Å^−^^3^

### Special details

**Table:**

Geometry. All e.s.d.'s (except the e.s.d. in the dihedral angle between two l.s. planes) are estimated using the full covariance matrix. The cell e.s.d.'s are taken into account individually in the estimation of e.s.d.'s in distances, angles and torsion angles; correlations between e.s.d.'s in cell parameters are only used when they are defined by crystal symmetry. An approximate (isotropic) treatment of cell e.s.d.'s is used for estimating e.s.d.'s involving l.s. planes.
Refinement. Refinement of *F*^2^ against ALL reflections. The weighted *R*-factor *wR* and goodness of fit *S* are based on *F*^2^, conventional *R*-factors *R* are based on *F*, with *F* set to zero for negative *F*^2^. The threshold expression of *F*^2^ > σ(*F*^2^) is used only for calculating *R*-factors(gt) *etc*. and is not relevant to the choice of reflections for refinement. *R*-factors based on *F*^2^ are statistically about twice as large as those based on *F*, and *R*-factors based on ALL data will be even larger.

### Fractional atomic coordinates and isotropic or equivalent isotropic displacement parameters (Å^2^)

**Table:**

	*x*	*y*	*z*	*U*_iso_*/*U*_eq_	
O1	0.37531 (7)	0.7195 (2)	0.60223 (11)	0.0223 (3)	
O2	0.31966 (8)	0.9210 (2)	0.49638 (12)	0.0279 (4)	
O3	0.10542 (9)	0.3305 (2)	0.65096 (12)	0.0339 (4)	
N1	0.27420 (9)	0.4903 (2)	0.50849 (12)	0.0211 (4)	
N2	0.17341 (10)	0.3084 (3)	0.52035 (13)	0.0267 (4)	
C1	0.43599 (11)	0.8207 (3)	0.46864 (15)	0.0212 (4)	
C2	0.49532 (11)	0.7190 (3)	0.50441 (16)	0.0238 (4)	
H2	0.4958	0.6552	0.5658	0.029*	
C3	0.55359 (11)	0.7105 (3)	0.45073 (16)	0.0246 (4)	
H3	0.5939	0.6414	0.4760	0.030*	
C4	0.55385 (11)	0.8024 (3)	0.35973 (16)	0.0237 (4)	
C5	0.49471 (12)	0.9054 (3)	0.32543 (16)	0.0254 (5)	
H5	0.4943	0.9695	0.2642	0.030*	
C6	0.43625 (11)	0.9158 (3)	0.37932 (16)	0.0235 (4)	
H6	0.3965	0.9877	0.3553	0.028*	
C7	0.61739 (12)	0.7901 (3)	0.30194 (17)	0.0277 (5)	
H7A	0.6324	0.6674	0.2988	0.041*	
H7B	0.6569	0.8599	0.3363	0.041*	
H7C	0.6042	0.8348	0.2331	0.041*	
C8	0.37095 (11)	0.8299 (3)	0.52067 (15)	0.0209 (4)	
C9	0.31180 (11)	0.6918 (3)	0.64461 (15)	0.0210 (4)	
C10	0.30198 (11)	0.7733 (3)	0.73413 (15)	0.0233 (4)	
H10	0.3360	0.8566	0.7634	0.028*	
C11	0.24158 (12)	0.7332 (3)	0.78214 (15)	0.0255 (5)	
H11	0.2340	0.7921	0.8428	0.031*	
C12	0.19361 (11)	0.6093 (3)	0.74151 (15)	0.0236 (4)	
H12	0.1533	0.5808	0.7746	0.028*	
C13	0.20422 (11)	0.5243 (3)	0.65079 (15)	0.0214 (4)	
C14	0.26278 (10)	0.5673 (3)	0.59976 (14)	0.0195 (4)	
C15	0.15613 (11)	0.3839 (3)	0.61094 (15)	0.0252 (5)	
C16	0.23027 (11)	0.3681 (3)	0.47275 (15)	0.0228 (4)	
C17	0.24100 (12)	0.2818 (3)	0.37470 (16)	0.0270 (5)	
H17A	0.2801	0.3402	0.3458	0.040*	
H17B	0.1968	0.2906	0.3273	0.040*	
H17C	0.2531	0.1581	0.3869	0.040*	
C18	0.12952 (13)	0.1602 (3)	0.47919 (16)	0.0311 (5)	
C19	0.15047 (12)	−0.0095 (3)	0.50999 (18)	0.0316 (5)	
H19	0.1922	−0.0288	0.5567	0.038*	
C20	0.10838 (14)	−0.1495 (3)	0.47021 (19)	0.0348 (5)	
H20	0.1219	−0.2665	0.4880	0.042*	
C21	0.04685 (13)	−0.1168 (4)	0.40471 (19)	0.0362 (6)	
H21	0.0177	−0.2127	0.3794	0.043*	
C22	0.02648 (14)	0.0491 (4)	0.37508 (18)	0.0350 (6)	
H22	−0.0158	0.0669	0.3291	0.042*	
C23	0.06853 (13)	0.1950 (4)	0.41299 (18)	0.0333 (5)	
C24	0.04695 (14)	0.3739 (4)	0.3848 (2)	0.0406 (6)	
H24A	0.0457	0.4443	0.4461	0.061*	
H24B	−0.0008	0.3725	0.3455	0.061*	
H24C	0.0814	0.4250	0.3437	0.061*	

### Atomic displacement parameters (Å^2^)

**Table:**

	*U*^11^	*U*^22^	*U*^33^	*U*^12^	*U*^13^	*U*^23^
O1	0.0183 (7)	0.0267 (8)	0.0224 (7)	−0.0018 (6)	0.0046 (5)	0.0041 (6)
O2	0.0240 (7)	0.0329 (9)	0.0279 (8)	0.0037 (6)	0.0072 (6)	0.0059 (6)
O3	0.0299 (8)	0.0486 (11)	0.0258 (8)	−0.0159 (7)	0.0138 (6)	−0.0061 (7)
N1	0.0214 (8)	0.0249 (9)	0.0181 (8)	−0.0008 (7)	0.0062 (6)	0.0012 (7)
N2	0.0267 (9)	0.0356 (11)	0.0194 (8)	−0.0107 (8)	0.0094 (7)	−0.0046 (7)
C1	0.0214 (10)	0.0221 (10)	0.0205 (9)	−0.0031 (8)	0.0043 (7)	−0.0025 (8)
C2	0.0239 (10)	0.0247 (11)	0.0233 (10)	−0.0016 (8)	0.0044 (8)	0.0014 (8)
C3	0.0232 (10)	0.0225 (11)	0.0286 (11)	0.0005 (8)	0.0054 (8)	0.0004 (8)
C4	0.0246 (10)	0.0235 (10)	0.0243 (10)	−0.0048 (8)	0.0083 (8)	−0.0056 (8)
C5	0.0290 (11)	0.0271 (11)	0.0209 (10)	−0.0024 (9)	0.0065 (8)	0.0012 (8)
C6	0.0230 (10)	0.0247 (10)	0.0227 (10)	−0.0004 (8)	0.0032 (8)	0.0008 (8)
C7	0.0283 (11)	0.0281 (11)	0.0286 (11)	−0.0006 (9)	0.0113 (9)	−0.0020 (9)
C8	0.0212 (9)	0.0223 (10)	0.0193 (9)	−0.0029 (8)	0.0030 (7)	−0.0009 (7)
C9	0.0189 (9)	0.0239 (10)	0.0209 (9)	0.0005 (8)	0.0057 (7)	0.0044 (8)
C10	0.0269 (10)	0.0209 (10)	0.0218 (10)	−0.0011 (8)	0.0015 (8)	0.0013 (8)
C11	0.0321 (11)	0.0273 (11)	0.0183 (9)	0.0010 (9)	0.0080 (8)	−0.0001 (8)
C12	0.0238 (10)	0.0286 (11)	0.0195 (9)	0.0006 (8)	0.0075 (8)	0.0018 (8)
C13	0.0205 (9)	0.0266 (10)	0.0178 (9)	−0.0003 (8)	0.0054 (7)	0.0024 (8)
C14	0.0201 (9)	0.0231 (10)	0.0159 (9)	0.0013 (8)	0.0040 (7)	0.0022 (7)
C15	0.0237 (10)	0.0344 (12)	0.0188 (9)	−0.0042 (9)	0.0074 (8)	−0.0003 (8)
C16	0.0241 (10)	0.0273 (11)	0.0183 (9)	−0.0028 (8)	0.0078 (8)	0.0016 (8)
C17	0.0309 (11)	0.0317 (12)	0.0203 (10)	−0.0060 (9)	0.0108 (8)	−0.0036 (8)
C18	0.0319 (12)	0.0415 (14)	0.0218 (10)	−0.0080 (10)	0.0102 (9)	−0.0064 (9)
C19	0.0276 (11)	0.0339 (13)	0.0347 (12)	−0.0023 (9)	0.0096 (9)	−0.0011 (10)
C20	0.0356 (12)	0.0355 (13)	0.0355 (12)	−0.0007 (10)	0.0131 (10)	0.0001 (10)
C21	0.0293 (12)	0.0482 (15)	0.0334 (12)	−0.0061 (11)	0.0128 (9)	−0.0019 (11)
C22	0.0357 (12)	0.0456 (14)	0.0254 (11)	0.0019 (11)	0.0106 (9)	−0.0053 (10)
C23	0.0307 (12)	0.0432 (14)	0.0276 (11)	−0.0010 (10)	0.0106 (9)	−0.0032 (10)
C24	0.0332 (13)	0.0548 (17)	0.0332 (13)	0.0001 (12)	0.0013 (10)	−0.0041 (12)

### Geometric parameters (Å, º)

**Table:**

O1—C8	1.368 (2)	C10—C11	1.407 (3)
O1—C9	1.401 (2)	C10—H10	0.9500
O2—C8	1.201 (3)	C11—C12	1.370 (3)
O3—C15	1.220 (3)	C11—H11	0.9500
N1—C16	1.296 (3)	C12—C13	1.407 (3)
N1—C14	1.390 (3)	C12—H12	0.9500
N2—C16	1.388 (3)	C13—C14	1.405 (3)
N2—C15	1.411 (3)	C13—C15	1.459 (3)
N2—C18	1.465 (3)	C16—C17	1.498 (3)
C1—C6	1.393 (3)	C17—H17A	0.9800
C1—C2	1.394 (3)	C17—H17B	0.9800
C1—C8	1.483 (3)	C17—H17C	0.9800
C2—C3	1.384 (3)	C18—C23	1.381 (3)
C2—H2	0.9500	C18—C19	1.400 (4)
C3—C4	1.400 (3)	C19—C20	1.394 (3)
C3—H3	0.9500	C19—H19	0.9500
C4—C5	1.391 (3)	C20—C21	1.381 (4)
C4—C7	1.505 (3)	C20—H20	0.9500
C5—C6	1.390 (3)	C21—C22	1.367 (4)
C5—H5	0.9500	C21—H21	0.9500
C6—H6	0.9500	C22—C23	1.421 (4)
C7—H7A	0.9800	C22—H22	0.9500
C7—H7B	0.9800	C23—C24	1.460 (4)
C7—H7C	0.9800	C24—H24A	0.9800
C9—C10	1.377 (3)	C24—H24B	0.9800
C9—C14	1.404 (3)	C24—H24C	0.9800
			
C8—O1—C9	116.36 (15)	C13—C12—H12	120.0
C16—N1—C14	117.56 (17)	C14—C13—C12	120.85 (19)
C16—N2—C15	122.04 (18)	C14—C13—C15	118.97 (18)
C16—N2—C18	120.91 (17)	C12—C13—C15	120.12 (18)
C15—N2—C18	117.04 (17)	N1—C14—C9	119.44 (17)
C6—C1—C2	119.51 (19)	N1—C14—C13	122.78 (18)
C6—C1—C8	117.80 (19)	C9—C14—C13	117.77 (18)
C2—C1—C8	122.68 (19)	O3—C15—N2	121.0 (2)
C3—C2—C1	120.2 (2)	O3—C15—C13	124.76 (19)
C3—C2—H2	119.9	N2—C15—C13	114.28 (17)
C1—C2—H2	119.9	N1—C16—N2	124.17 (18)
C2—C3—C4	120.9 (2)	N1—C16—C17	119.07 (18)
C2—C3—H3	119.6	N2—C16—C17	116.75 (18)
C4—C3—H3	119.6	C16—C17—H17A	109.5
C5—C4—C3	118.45 (19)	C16—C17—H17B	109.5
C5—C4—C7	121.5 (2)	H17A—C17—H17B	109.5
C3—C4—C7	120.1 (2)	C16—C17—H17C	109.5
C6—C5—C4	121.1 (2)	H17A—C17—H17C	109.5
C6—C5—H5	119.5	H17B—C17—H17C	109.5
C4—C5—H5	119.5	C23—C18—C19	123.1 (2)
C5—C6—C1	119.9 (2)	C23—C18—N2	118.2 (2)
C5—C6—H6	120.0	C19—C18—N2	118.7 (2)
C1—C6—H6	120.0	C20—C19—C18	118.2 (2)
C4—C7—H7A	109.5	C20—C19—H19	120.9
C4—C7—H7B	109.5	C18—C19—H19	120.9
H7A—C7—H7B	109.5	C21—C20—C19	119.4 (2)
C4—C7—H7C	109.5	C21—C20—H20	120.3
H7A—C7—H7C	109.5	C19—C20—H20	120.3
H7B—C7—H7C	109.5	C22—C21—C20	122.1 (3)
O2—C8—O1	122.40 (18)	C22—C21—H21	118.9
O2—C8—C1	125.79 (19)	C20—C21—H21	118.9
O1—C8—C1	111.81 (17)	C21—C22—C23	120.0 (2)
C10—C9—O1	119.68 (18)	C21—C22—H22	120.0
C10—C9—C14	121.38 (18)	C23—C22—H22	120.0
O1—C9—C14	118.63 (18)	C18—C23—C22	117.1 (2)
C9—C10—C11	120.0 (2)	C18—C23—C24	121.7 (2)
C9—C10—H10	120.0	C22—C23—C24	121.2 (2)
C11—C10—H10	120.0	C23—C24—H24A	109.5
C12—C11—C10	120.06 (19)	C23—C24—H24B	109.5
C12—C11—H11	120.0	H24A—C24—H24B	109.5
C10—C11—H11	120.0	C23—C24—H24C	109.5
C11—C12—C13	119.92 (19)	H24A—C24—H24C	109.5
C11—C12—H12	120.0	H24B—C24—H24C	109.5
			
C6—C1—C2—C3	−1.0 (3)	C12—C13—C14—C9	2.7 (3)
C8—C1—C2—C3	177.69 (19)	C15—C13—C14—C9	−174.38 (18)
C1—C2—C3—C4	−0.4 (3)	C16—N2—C15—O3	179.2 (2)
C2—C3—C4—C5	1.2 (3)	C18—N2—C15—O3	−2.1 (3)
C2—C3—C4—C7	−179.4 (2)	C16—N2—C15—C13	−1.9 (3)
C3—C4—C5—C6	−0.7 (3)	C18—N2—C15—C13	176.80 (19)
C7—C4—C5—C6	180.0 (2)	C14—C13—C15—O3	176.9 (2)
C4—C5—C6—C1	−0.7 (3)	C12—C13—C15—O3	−0.3 (3)
C2—C1—C6—C5	1.5 (3)	C14—C13—C15—N2	−2.0 (3)
C8—C1—C6—C5	−177.18 (19)	C12—C13—C15—N2	−179.15 (19)
C9—O1—C8—O2	12.0 (3)	C14—N1—C16—N2	−0.8 (3)
C9—O1—C8—C1	−167.83 (17)	C14—N1—C16—C17	−179.84 (18)
C6—C1—C8—O2	−5.0 (3)	C15—N2—C16—N1	3.5 (3)
C2—C1—C8—O2	176.3 (2)	C18—N2—C16—N1	−175.1 (2)
C6—C1—C8—O1	174.84 (17)	C15—N2—C16—C17	−177.4 (2)
C2—C1—C8—O1	−3.8 (3)	C18—N2—C16—C17	4.0 (3)
C8—O1—C9—C10	−103.9 (2)	C16—N2—C18—C23	−92.2 (3)
C8—O1—C9—C14	82.5 (2)	C15—N2—C18—C23	89.1 (3)
O1—C9—C10—C11	−173.89 (18)	C16—N2—C18—C19	88.9 (3)
C14—C9—C10—C11	−0.4 (3)	C15—N2—C18—C19	−89.7 (3)
C9—C10—C11—C12	1.9 (3)	C23—C18—C19—C20	1.3 (3)
C10—C11—C12—C13	−1.1 (3)	N2—C18—C19—C20	−179.9 (2)
C11—C12—C13—C14	−1.3 (3)	C18—C19—C20—C21	−1.9 (3)
C11—C12—C13—C15	175.8 (2)	C19—C20—C21—C22	1.8 (4)
C16—N1—C14—C9	175.77 (19)	C20—C21—C22—C23	−0.9 (4)
C16—N1—C14—C13	−3.4 (3)	C19—C18—C23—C22	−0.5 (3)
C10—C9—C14—N1	178.96 (19)	N2—C18—C23—C22	−179.30 (19)
O1—C9—C14—N1	−7.5 (3)	C19—C18—C23—C24	178.1 (2)
C10—C9—C14—C13	−1.9 (3)	N2—C18—C23—C24	−0.7 (3)
O1—C9—C14—C13	171.66 (17)	C21—C22—C23—C18	0.3 (3)
C12—C13—C14—N1	−178.11 (18)	C21—C22—C23—C24	−178.3 (2)
C15—C13—C14—N1	4.8 (3)		

### Hydrogen-bond geometry (Å, º)

Cg1 is the centroid of the C18–C23 benzene ring.

**Table:**

*D*—H···*A*	*D*—H	H···*A*	*D*···*A*	*D*—H···*A*
C17—H17*C*···O2^i^	0.98	2.55	3.434 (3)	150
C21—H21···O3^ii^	0.95	2.47	3.299 (3)	146
C12—H12···*Cg*1^iii^	0.95	2.79	3.658 (2)	153

Symmetry codes: (i) *x*, *y*−1, *z*; (ii) −*x*, −*y*, −*z*+1; (iii) *x*, −*y*+1/2, *z*+1/2.
